# Allelic variant in *SLC6A3* rs393795 affects cerebral regional homogeneity and gait dysfunction in patients with Parkinson’s disease

**DOI:** 10.7717/peerj.7957

**Published:** 2019-11-04

**Authors:** Lina Wang, Yongsheng Yuan, Jianwei Wang, Yuting Shen, Yan Zhi, Junyi Li, Min Wang, Kezhong Zhang

**Affiliations:** 1 Department of Neurology, The First Affiliated Hospital of Nanjing Medical University, Nanjing, China; 2 Department of Radiology, The First Affiliated Hospital of Nanjing Medical University, Nanjing, China

**Keywords:** Parkinson’s disease, SLC6A3, rs393795, Imaging genetics, Regional homogeneity, Gait dysfunction

## Abstract

**Aims:**

We sought to explore the role of the *SLC6A3* rs393795 allelic variant in cerebral spontaneous activity and clinical features in Parkinson’s disease (PD) via imaging genetic approach.

**Methods:**

Our study recruited 50 PD and 45 healthy control (HC) participants to provide clinical, genetic, and resting state functional magnetic resonance imaging (rs-fMRI) data. All subjects were separated into 16 PD-AA, 34 PD-CA/CC, 14 HC-AA, and 31 HC-CA/CC four subgroups according to *SLC6A3* rs393795 genotyping. Afterwards, main effects and interactions of groups (PD versus HC) and genotypes (AA versus CA/CC) on cerebral function reflected by regional homogeneity (ReHo) were explored using two-way analysis of covariance (ANCOVA) after controlling age and gender. Finally, Spearman’ s correlations were employed to investigate the relationships between significantly interactive brain regions and clinical manifestations in PD subgroups.

**Results:**

Compared with HC subjects, PD patients exhibited increased ReHo signals in left middle temporal gyrus and decreased ReHo signals in left pallidum. Compared with CA/CC carriers, AA genotype individuals showed abnormal increased ReHo signals in right inferior frontal gyrus (IFG) and supplementary motor area (SMA). Moreover, significant interactions (affected by both disease factor and allelic variation) were detected in right inferior temporal gyrus (ITG). Furthermore, aberrant increased ReHo signals in right ITG were observed in PD-AA in comparison with PD-CA/CC. Notably, ReHo values in right ITG were negatively associated with Tinetti Mobility Test (TMT) gait subscale scores and positively related to Freezing of Gait Questionnaire (FOG-Q) scores in PD-AA subgroup.

**Conclusions:**

Our findings suggested that *SLC6A3* rs393795 allelic variation might have a trend to aggravate the severity of gait disorders in PD patients by altering right SMA and IFG function, and ultimately result in compensatory activation of right ITG. It could provide us with a new perspective for exploring deeply genetic mechanisms of gait disturbances in PD.

## Introduction

Parkinson’s disease (PD), characterized by gradually aggravated nigrostriatal dopaminergic neuronal dysfunction, is a progressive neurodegenerative disorder (Fearnley & Lees, 1991). The pathophysiological process of PD is influenced not only by nongenetic factors such as environmental risks and aging but also by genetic polymorphisms (Raza, Anjum & Shakeel, 2019). Recently, the role of associative gene polymorphisms in sporadic PD has increasingly been investigated. The human dopamine transporter (DAT) gene, now known as *SLC6A3* (chromosome 5p15.3), is one of the most crucial candidate genes in sporadic PD due to its role in dopamine (DA) neuron function (Zhai et al., 2014). *SLC6A3* encodes DAT, which is a membrane protein located in the presynaptic region of dopaminergic neurons, where it can rapidly transport DA from the extracellular space into the cytosol of the presynaptic neuron, contributing to overall DA neurotransmission in brain (Cragg & Rice, 2004; Lohr et al., 2017). Recently, a large prospective trial from the Parkinson Progression Marker Initiative (PPMI) believed that the dopaminergic denervation in presynaptic striatum could be a bad predictor for the later development of freezing of gait (FOG) in almost 400 early PD patients (Kim et al., 2018). Moreover, a pharmacogenetic study suggested that *SLC6A3* polymorphisms appeared to be associated with the response to levodopa observed in advanced PD patients with gait disorders (Moreau et al., 2015). Hence, these studies confirmed the important role of *SLC6A3* polymorphisms in PD progression. However, in *SLC6A3*, most previous literature focused on the role of variable number tandem repeat (VNTR) polymorphism in the 3′  untranslated region in PD (Kim et al., 2000; Kang, Palmatier & Kidd, 1999). Recently, growing attention was paid to other single nucleotide polymorphisms (SNPs), such as rs393795. Moreover, several latest studies indicated that *SLC6A3* rs393795 played an important role in PD (Purcaro et al., 2019; Kaplan et al., 2014). However, the *SLC6A3* rs393795 polymorphisms were not widely investigated in PD, particularly about how the variant affected cerebral spontaneous neuronal activity and clinical manifestations in PD. Therefore, we employed this study to investigate the effects of the *SLC6A3* rs393795 allelic variant on the brain spontaneous neuronal activity and relevant clinical features in PD.

Imaging genetics was applied in our research, which could provide us with new insights into the influence of genetic variant on brain function (Bogdan et al., 2017). The method has been widely employed to explore several neurological and psychiatric diseases, such as Alzheimer’s disease (AD) (Apostolova et al., 2018). Actually, resting-state functional magnetic resonance imaging (rs-fMRI) can reveal the brain spontaneous neuronal activity by examining spontaneous fluctuations in the blood oxygen level dependent (BOLD) signal at rest (Prodoehl, Burciu & Vaillancourt, 2014). So, it makes it possible to explore the probable role of gene polymorphisms in brain activity. Calculating the similarity of voxel fluctuations within a given cluster, regional homogeneity (ReHo) can embody the local synchronization of spontaneous BOLD signals (Long et al., 2008). This approach has been widely adopted in PD studies, and even was considered as a potential diagnostic biomarker for PD (Li et al., 2016). However, few researchers focused on the probable role of gene polymorphisms in brain region dysfunction in PD. Thus, we employed this study to explore the role of *SLC6A3* rs393795 allelic variant in cerebral spontaneous neuronal activity and relevant clinical presentations in PD, by examining cerebral ReHo signal alterations as well as the correlations between altered brain interactive regional ReHo values and clinical symptoms.

## Materials & Methods

### Subjects

Our study enrolled 50 PD patients and 45 healthy control (HC) participants, which were initially clinically evaluated by Kezhong Zhang, a neurologist expert in movement disorders, especially in PD. Inclusion criteria were as follows: (1) meeting the diagnostic criteria for idiopathic PD according to the United Kingdom Parkinson’s Disease Society Brain Bank criteria (Hughes et al., 1992); (2) having no medical history of stroke, brain tumor, traumatic brain injury, dementia or psychiatric disorders; (3) without contraindications for MRI scans; (4) not taking sedative and hypnotic medications.

Subjects were evaluated by face-to-face interviews and clinical associative scales. Unified Parkinson’s Disease Rating Scale section III (UPDRS-III) (Goetz, 2003), Tinetti Mobility Test (TMT) (Kegelmeyer et al., 2007), Freezing of Gait Questionnaire (FOG-Q) (Tambasco et al., 2015) and Timed Up and Go (TUG) test (Chantanachai, Pichaiyongwongdee & Jalayondeja, 2014) were performed to detect motor symptoms in PD. Moreover, Hamilton Anxiety Rating Scale (HAMA) (Kummer, Cardoso & Teixeira, 2010), 17-item Hamilton Rating Scale for Depression (HAMD-17) (Worboys, 2013), Apathy Scale (AS) (Leentjens et al., 2008), Epworth Sleeping Scale (ESS) (Schrempf et al., 2014) and Fatigue Severity Scale (FSS) (Valderramas, Feres & Melo, 2012) were used to evaluate the non-motor manifestations in PD. In addition, we calculated the total levodopa equivalent daily dose (LEDD), LEDD of levodopa preparations and LEDD of dopamine receptor agonists for each PD individual (Tomlinson et al., 2010).

To reduce the pharmacological effects on neural activity, clinical assessments and MRI scans were performed at least 12 h after withdrawal from drugs for all PD participants. Meanwhile, age, gender, education and ethnicity matched HC subjects were enrolled in this study to eliminate other neurological and psychological disorders or neuroimaging disturbances. In addition, fasting peripheral blood samples from all participants were collected for genetic assessment. Each subject agreed to join our study and signed an informed consent form. Our study was approved by the Ethics Committee of the First Affiliated Hospital of Nanjing Medical University (2014-SRFA-097).

### Genotyping

Fasting peripheral venous blood samples were obtained from all consenting participants after at least 12 h after withdrawal from drugs and foods in the morning. All blood samples were randomly numbered and genotyped by a “blinded” independent researcher. Each subject’s genomic DNA was extracted using a DNA direct kit (BioTeKe Corpration, Beijing, China). The *SLC6A3* rs393795 data were obtained and analyzed by the Beijing Genomics Institute (BGI) using MassARRAY TYPER 4.0 software (Agena Bioscience, San Diego, CA, USA). According to the manufacturer’s instructions, a series of experimental procedures were strictly carried out, including DNA isolation, polymerase chain reaction (PCR) amplification, shrimp alkaline phosphatase (SAP) treatment, addition to SpectroCHIP bioarray and matrix-assisted laser desorption ionisation time-of-flight mass spectrometry analysis. Afterwards, all participants were further divided into four different subgroups (PD-AA, PD-CA/CC, HC-AA, and HC-CA/CC) in agreement with previous genetic studies (van Munster et al., 2010a; van Munster et al., 2010b).

### Magnetic resonance imaging Acquisition

MRI was performed on a 3.0 T Siemens MAGNETOM Verio whole-body MRI scanner (Siemens Medical Solutions, Munich, Germany) with eight-channel phase-array head coils. During the resting experiments, we adopted tight foam to fix head and ear-plugs to reduce noise. Falling asleep, thinking about anything and movements were not advised, when participants were relaxed with their eyes closed. Whole brain anatomical images were obtained for all individuals. Scan parameters were as follows: repetition time (TR)/echo time (TE) = 1900/2.95 ms, flip angle = 9°, field of view (FOV) = 230 × 230 mm^2^, matrix size = 256 × 256, voxel size = 1 × 1 × 1 mm^3^, slice thickness = one mm, and number of slices = 160. Whole brain rs-fMRI images were gained with an echo-planar imaging (EPI) sequence. Scan parameters were as follows: TR/TE = 2,000/21 ms, flip angle = 90°, FOV = 256 × 256 mm^2^, in-plane matrix = 64 × 64, slice thickness = three mm, number of slices = 35, no slice gap, voxel size = 3 × 3 × 3 mm^3^, and total volumes = 240.

### Resting-state fMRI Data Processing and ReHo Acquisition

Data preprocessing was performed using the Data Processing Assistant for rs-fMRI (DPARSF: http://www.restfmri.net/forum/DPARSF) based on Statistical Parametric Mapping (SPM: http://www.fil.ion.ucl.ac.uk/spm/). Image data were analyzed with SPM and Resting-State fMRI Data Analysis Tookit (REST: http://www.restfmri.net). Removed the first 10 time points to reduce transient signal changes caused by unstable magnetic field and to permit subjects to be accustomed to the scanning circumstance. Preprocessing included standard slice timing, head motion correction, realignment, spatial normalization by diffeomorphic anatomical registration through exponentiated lie algebra (DARTEL; voxel size [3, 3, 3]). Additionally, nuisance signal removal via multiple regression adjusting for white matter, cerebrospinal fluid, head motion parameters, and 0.01–0.08 Hz pass filtering were applied to reduce signal to noise ratios. Individuals with more than 2.0 mm or 2-degree cumulative translation or rotation head motion were excluded from our study.

ReHo maps were accomplished in a voxel-wise way via calculating Kendall’s coefficient of concordance (KCC, also called ReHo signal) between time series of a given voxel with its nearest neighbors (26 voxels). In order to reduce individual differences, ReHo maps was normalized by dividing the KCC for each voxel by the average KCC of the whole brain. Ultimately, a four mm full width at half-maximum (FWHM) Gaussian filter was adopted to smooth the data in order to suppress noise and effects caused by residual differences in functional and rotational anatomy during inter-subject averaging period.

### Statistical analysis

Demographic and clinical characteristics of participants in different groups were compared using SPSS 20.0 statistical analysis software (SPSS Inc. Chicago, IL, USA). Actually, chi-square test, one-way analysis of variance (ANOVA), and Kruskal-Wallis test were applied to test gender, age, and education difference among the four subgroups, respectively. Other clinical features were analyzed by Mann–Whitney tests between PD two subgroups due to the non-normality of data distribution. Besides, the Hardy-Weinberg Equilibrium (HWE) of the genotype frequencies was analyzed by chi-square test. *P* < 0.05 was considered significant.

Two-way factorial analysis of covariance (ANCOVA: groups × genotypes; groups: PD versus HC; genotypes: AA versus CA/CC) was employed to examine significantly different brain clusters, adjusting for age and gender (voxel-wise *p* < 0.001 and cluster size >11 voxels, corrected by AlphaSim program in the REST software). Afterwards, post hoc tests were conducted to explore further statistical differences.

Finally, Spearman’s correlations were applied to the examine the relationships between the ReHo values extracted from the significantly interactive regions and clinical symptoms in PD, respectively (*p* < 0.05).

## Results

### Clinical features

The demographic and clinical characteristics of all participants (*n* = 95) were presented in Table 1. All individuals were of Han Chinese descent. No significant differences were found in gender, age and education among the four subgroups. Moreover, PD-AA and PD-CA/CC groups exhibited similar disease duration, H&Y stage, LEDD, LEDD of levodopa preparations and LEDD of dopamine receptor agonists, UPDRS-III, Total TMT, TMT balance subscale, TMT gait subscale, FOG-Q and TUG test scores. No significant differences were observed in HAMA, HAMD-17, AS, ESS and FSS scores as well. In addition, 40 of 50 (80%) PD patients were taking dopaminergic agents; of those, 38 of 50 (76%) took levodopa, 29 of 50 (58%) dopamine receptor agonists, and 26 of 50 (52%) both agents.

**Table 1:** Demographic and clinical characteristics of all subjects.

Variables	PD-AA	PD-CA/CC	HC-AA	HC-CA/CC	*P* value
*n*	16	34	14	31	NA
Gender (M/F)	11/5	23/11	9/5	20/11	0.987^a^
Age (y)	65.13 ± 10.11	66.15 ± 8.36	65.43 ± 4.50	62.26 ± 4.75	0.176^b^
Education (y)	10.94 ± 3.30	11.88 ± 3.45	11.64 ± 3.46	11.55 ± 3.54	0.849^c^
Disease duration (y)	3.71 ± 4.86	4.75 ± 3.18	NA	NA	0.052^d^
H&Y stage	2.06 ± 0.77	2.18 ± 0.67	NA	NA	0.625^d^
LEDD (mg/d)	410.94 ± 300.65	386.31 ± 300.83	NA	NA	0.708^d^
Levodopa preparations (mg/d)	254.69 ± 185.79	272.72 ± 202.37	NA	NA	0.846^d^
Dopamine receptor agonists (mg/d)	33.59 ± 41.26	42.61 ± 44.18	NA	NA	0.463^d^
UPDRS-III	22.63 ± 9.34	21.76 ± 8.96	NA	NA	0.950^d^
Total TMT	22.63 ± 4.46	21.79 ± 6.01	NA	NA	0.942^d^
TMT balance subscale	13.81 ± 2.51	12.79 ± 3.59	NA	NA	0.409^d^
TMT gait subscale	8.81 ± 2.48	9.00 ± 2.79	NA	NA	0.659^d^
FOG-Q	5.81 ± 5.15	5.41 ± 5.89	NA	NA	0.599^d^
TUG (s)	14.13 ± 4.08	15.12 ± 6.85	NA	NA	0.815^d^
HAMD-17	7.00 ± 5.18	5.06 ± 3.85	NA	NA	0.191^d^
HAMA	10.19 ± 6.76	8.29 ± 4.67	NA	NA	0.538^d^
AS	16.44 ± 7.96	15.76 ± 7.75	NA	NA	0.786^d^
ESS	4.69 ± 2.57	5.71 ± 4.32	NA	NA	0.699^d^
FSS	34.33 ± 17.75	26.41 ± 14.47	NA	NA	0.140^d^

**Notes.**

Data are presented as mean values ± SD.

Abbreviations PD-AA Parkinson’s disease with AA homozygous carries PD-CA/CC Parkinson’s disease with C allele carries HC-AA healthy control with AA homozygous carries HC-CA/CC healthy control with C allele carries M Male F Female y year H&Y Hoehn and Yahr stage LEDD Levodopa equivalent daily dose UPDRS Unified Parkinson’s disease rating scale TMT Tinetti Mobility Test FOG-Q Freezing of Gait Questionnaire TUG Timed Up and Go HAMD-17 17-item Hamilton Rating Scale for Depression HAMA Hamilton Anxiety Rating Scale AS Apathy Scale ESS Epworth Sleeping Scale FSS Fatigue Severity Scale NA Not applicable

* *p* < 0.05 was considered significant.

a chi-square test.

b One-way analysis of variance.

c Kruskal–Wallis test.

d Mann–Whitney test.

### Hardy–Weinberg equilibrium

The Table 2 showed that the distribution of genotype frequencies of the candidate SNP *SLC6A3* rs393795 was in HWE (PD: *χ*^2^ = 1.974, *p* = 0.160; HC: *χ*^2^ = 1.867, *p* = 0.172).

**Table 2:** Genotype frequencies for SLC6A3 rs393795 in PD and HC groups.

Genotypes	PD	HC	Total
AA	16 (32%)	14 (31%)	30 (32%)
CA	20 (40%)	26 (58%)	46 (48%)
CC	14 (28%)	5(11%)	19 (20%)

**Notes.**

Genotype frequencies for SLC6A3 rs393795 in PD group (*χ*^2^ = 1.974, *p* = 0.160) and HC group (*χ*^2^ = 1.867, *p* = 0.172) didn’t deviate from Hardy–Weinberg equilibrium.

Abbreviations PD Parkinson’s disease HC Healthy control

### Regional homogeneity

**Figure 1:**
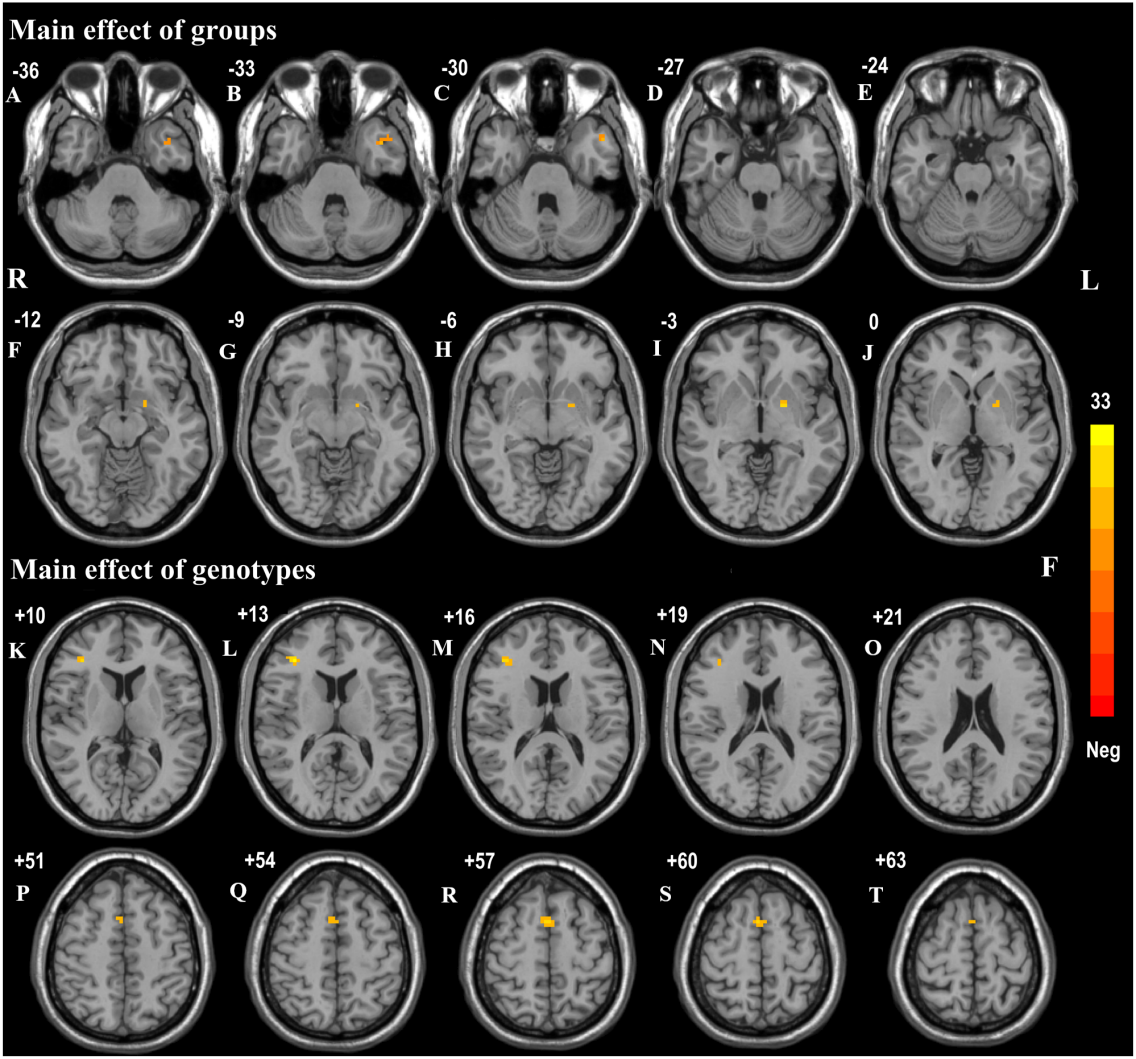
Main effects of groups and genotypes in all participants. (A–J) Main effect of diagnostic groups (PD versus HC) on ReHo was shown in left middle temporal gyrus and left pallidum in all participants; (K–T): Main effect of genotypes (AA versus CA/CC) on ReHo was observed in right IFG and SMA in all individuals. These findings were obtained via two-way factorial analysis of covariance (ANCOVA: groups × genotypes; groups: PD versus HC; genotypes: AA versus CA/CC), adjusting for age and gender. Thresholds were set at a corrected *p* < 0.001, determined by Monte Carlo simulation. The color bar indicated the *F* values from ANCOVA. Abbreviations: ReHo, Regional homogeneity; PD, Parkinson’s disease; HC, healthy control; IFG, inferior frontal gyrus; SMA, supplementary motor area; R, right; L, left.

**Figure 2:**
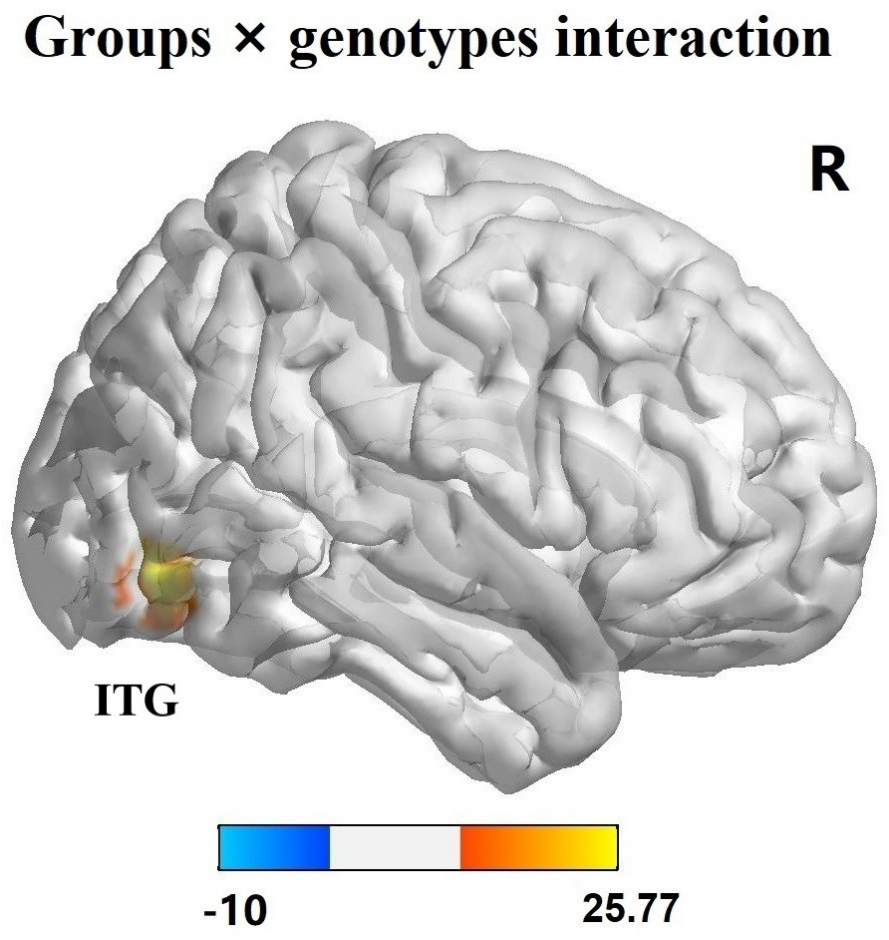
Interaction analysis of groups and genotypes. Significant interaction of groups and genotypes was found in right ITG by two-way factorial analysis of covariance (ANCOVA), adjusting for age and gender. The color bar presented the *F* values from ANCOVA. Abbreviations: ITG, inferior temporal gyrus; R, right.

The effects of diagnostic groups (PD versus HC) and genotypes (AA versus CA/CC) on ReHo values analyzed by two-way ANCOVA were shown in Table 3. The significant main effect of groups (PD versus HC, regardless of allelic variant status) was observed in left middle temporal gyrus (*F* = 17.91, *p* < 0.001, corrected) and pallidum (*F* = 22.66, *p* < 0.001, corrected) (Figs. 1A–1J). Furthermore, PD patients exhibited increased ReHo signals in left middle temporal gyrus but reduced ReHo signals in left pallidum in comparison with HC subjects. The significant main effect of genotypes (AA versus CA/CC carriers, regardless of disease status) was discovered in right inferior frontal gyrus (IFG, *F* = 32.73, *p* < 0.001, corrected) and supplementary motor area (SMA, *F* = 22.21, *p* < 0.001, corrected) (Figs. 1K–1T). Besides, AA genotype participants showed aberrant increased ReHo signals in right IFG and SMA compared with CA/CC carriers. It was worth noting that significant interactions between groups and genotypes (affected by both disease factor and allelic variation) were found in the right inferior temporal gyrus (ITG, *F* = 25.10, *p* < 0.001, corrected) (Fig. 2). Hereafter, post hoc tests (Bonferroni) were employed to explore further interactive differences, and *p* < 0.008 (0.05/6 [all possible pair-wise comparisons]) was considered significant. Specially, abnormal increased ReHo signals in right ITG were observed in PD-AA compared with PD-CA/CC group (*p* < 0.001), while reduced ReHo signals in right ITG were detected in AA homozygotes in comparison with CA/CC carriers in HC subgroups (*p* = 0.004).

**Table 3:** Groups × genotypes ANCOVA of ReHo.

Brain regions (AAL)	Peak MNI Coordinates x, y, z (mm)			Peak *F* value	Cluster size (voxels)
(1) Main effect of groups					
Temporal_Mid_L	−39	6	−36	17.91	14
Pallidum_L	−18	0	−3	22.66	12
(2) Main effect of genotypes					
Frontal_Inf_R	36	33	12	32.73	13
Supp_Motor_Area_R	3	9	57	22.21	12
(3) Groups × genotypes interaction					
Temporal_Inf_R	57	−66	−9	25.10	21

**Notes.**

Two-way factorial analysis of covariance (ANCOVA: groups × genotypes; groups: PD versus HC, genotypes: AA versus CA/CC) was performed, adjusting for age and gender. A corrected threshold by Monte Carlo simulation was set at *P* < 0.001.

Abbreviations PD Parkinson disease HC Healthy control ReHo Regional homogeneity AAL Anatomical automatic labeling MNI Montreal Neurological Institute R right L left

### Correlation analysis

**Figure 3:**
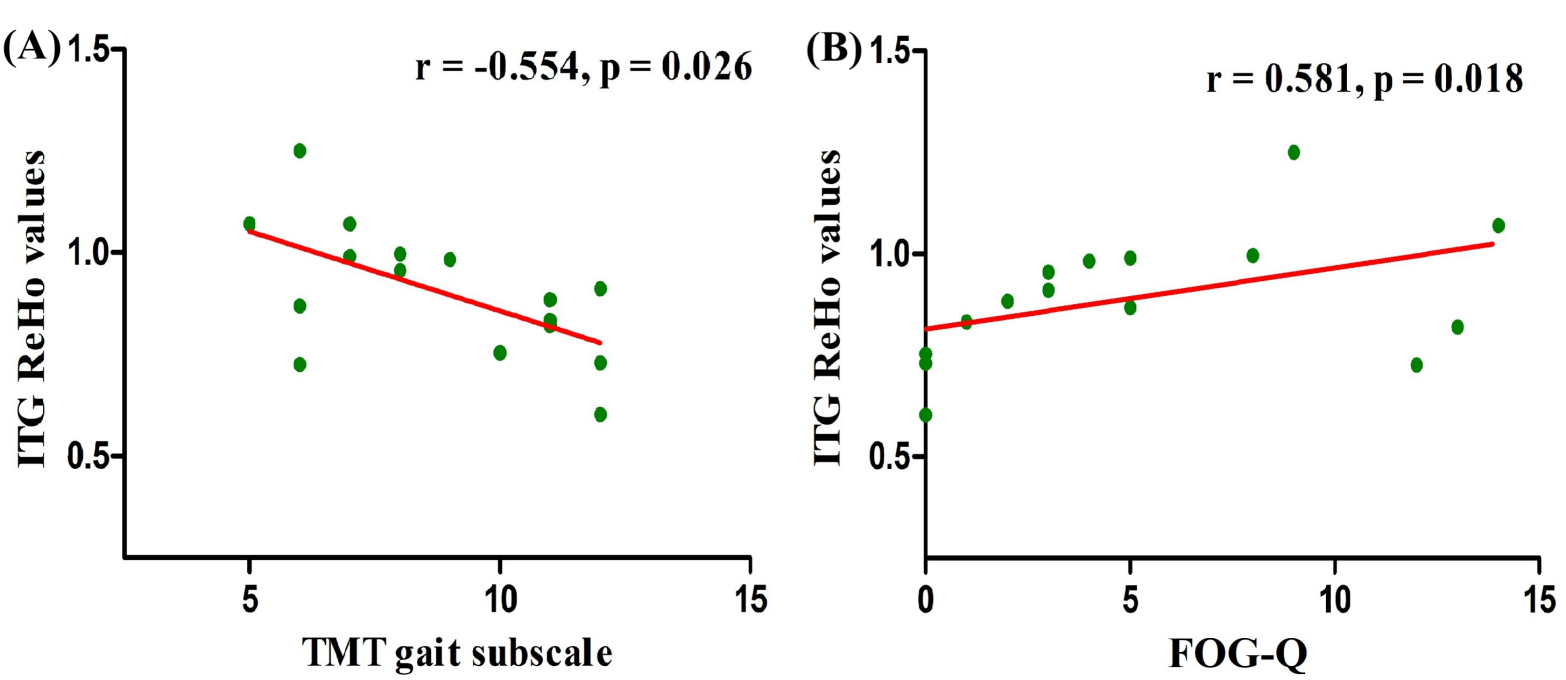
Correlation analysis between gait assessments and ReHo values in right ITG among PD-AA carriers. ReHo values in right ITG affected by the interaction between groups and genotypes, were negatively associated with TMT gait subscale scores (*r* =  − 0.554, *p* = 0.026) (A) and positively related to FOG-Q scores (*r* = 0.581, *p* = 0.018) (B) in PD individuals carrying AA. The associations were investigated by Spearman’s correlation. Abbreviations: ReHo, Regional homogeneity; PD, Parkinson’s disease; ITG, inferior temporal gyrus; TMT, Tinetti Mobility Test; FOG-Q, Freezing of Gait Questionnaire.

Correlations between the clinical scores and ReHo values extracted from significant interactive clusters were investigated in PD patients. ReHo values in right ITG were negatively associated with TMT gait subscale scores (*r* =  − 0.554, *p* = 0.026) (Fig. 3A) and positively related to FOG-Q scores (*r* = 0.581, *p* = 0.018) (Fig. 3B) in PD patients with AA genotype. These results suggested that the severity of gait disorders in PD was closely associated with altered right ITG functional activity modulated by the AA genotype of *SLC6A3* rs393795. Nevertheless, in the two subgroups of PD, no other significant correlations were found between right ITG ReHo values and any other clinical scores (*p* > 0.05).

## Discussion

In this imaging genetic study, we explored the role of *SLC6A3* rs393795 allelic variant in brain regional homogeneous activity in sporadic PD patients. Compared with CA/CC carriers, AA genotype individuals showed abnormal increased ReHo signals in right IFG and SMA in all participants. Moreover, our interaction investigation revealed that AA homozygotes exhibited aberrant increased ReHo signals in right ITG compared with CA/CC carriers in PD subgroups. Notably, ReHo values in right ITG were negatively associated with TMT gait subscale scores and positively related to FOG-Q scores in PD-AA group.

*SLC6A3* rs393795 was supposed to be the candidate SNP for sporadic PD, supported by numerous imaging and pharmacogenetic studies validating the close relationship between DAT and PD (Djaldetti et al., 2018; Kim et al., 2018). Particularly, the depletion of presynaptic DA was believed to be closely connected with gait disorders in PD, and even could be a bad prognostic sign in terms of FOG development (Kim et al., 2018; Djaldetti et al., 2018). Moreover, the rs393795 SNP (located on intron 4) falls within the same linkage disequilibrium cluster with rs460000 SNP (located within the exon 4-intron 3 boundary) (Cartegni, Chew & Krainer, 2002), which has been associated with frontostriatal response inhibition circuits (Cummins et al., 2012). Consistent with this, our study showed that the SNP AA genotype effected the brain functional activation of right SMA and IFG in all individuals. Furthermore, the right SMA and IFG, involved in frontal cortico-basal ganglia motor network, had been shown to participate in the functional organization of gait initiation or FOG phenomenon (Bartels & Leenders, 2008). Thus, we hypothesized that AA genotype could affect gait in human via altering the spontaneous activity of right SMA and ITG. However, no significant differences were discovered in gait associative scales between PD-AA and PD-CA/CC groups. These findings perhaps implied that the effects of the SNP polymorphisms were compensated and consequently not sufficient to cause significant different clinical manifestations. In good agreement with this, abnormal increased ReHo values in right SMA and IFG were observed in AA carriers compared with CA/CC carriers in our study. Alternatively, the *SLC6A3* rs393795 allelic variant might just play a regulatory instead of causative role in PD patients with gait disorders, in other words, not anyone with allelic variation will markedly develop or aggravate PD gait dysfunction. Consistent with this, *SLC6A3* rs393795 variant, falling within the same linkage disequilibrium cluster with two of the SNPs (rs458609, rs457702) and with rs460000 SNP, was merely indicated to participate in DAT translations by affecting alternative splicing of the DAT to some extend (Cartegni, Chew & Krainer, 2002; Talkowski et al., 2010). However, these didn’t represent our study worthless. On the contrary, it could deepen our understanding of genetic mechanisms underlying gait disorders in PD, contributing to more effective and precise diagnosis and treatments in the future.

Since both PD and *SLC6A3* rs393795 variant had effects on the dysfunction of frontal cortico-basal ganglia motor pathway, their interactions might lead to greater alterations in related brain regions. In good agreement with this, our interaction investigation revealed that AA carriers showed aberrant increased ReHo signals in right ITG in comparison with CA/CC carriers in PD groups. ITG was one of the key hubs in ventral visual pathway (Weller, 1988), which was characterized to support the processing of object quality or identity (Kravitz et al., 2013) and mainly dominated by right-hemisphere (Woolley et al., 2010). It was worth noting that a large amount of evidence indicated the effectiveness of various visual aids in compensating for gait difficulties in PD (Davidsdottir, Cronin-Golomb & Lee, 2005; Lee et al., 2012). Moreover, more and more literature demonstrated that gait was not only an automated motor activity, but also one increasingly dependent on external information, especially visual cues (Uc et al., 2005; Sage & Almeida, 2010). Additionally, dysfunction of right occipitotemporal “visual” networks was also detected in PD patients with gait deficits from different research populations (Tessitore et al., 2012; Wang et al., 2016). Hence, abnormal increased ReHo signals in right ITG observed in PD-AA group could be due to its compensatory activation when PD patients had a worse trend of gait disorders caused by a worse dysfunction of frontal cortico-basal ganglia motor network. Consistently, our further correlation analysis displayed that ReHo values in right ITG were negatively related to TMT gait subscale scores and positively associated with FOG-Q scores in PD-AA group. TMT gait subscale and FOG-Q both showed excellent validity and reliability for the valuation of different gait characteristics and had been widely used in PD researches (Kegelmeyer et al., 2007; Tambasco et al., 2015; Park et al., 2018; Li et al., 2018). Besides, the lower scores of TMT gait subscale represents more serious gait deficits, in contrast, the higher scores of FOG-Q means more serious FOG. Taken together, *SLC6A3* rs393795 AA genotype might have a tendency to aggravate the severity of gait dysfunction in PD by altering the SMA and IFG spontaneous activity, and ultimately lead to the compensatory activation of right ITG.

Nevertheless, AA homozygous of *SLC6A3* rs393795 was only previously reported to protect the elderly from delirium possibly by reducing the concentrations of DA in the brain (van Munster et al., 2010a; van Munster et al., 2010b). Meanwhile, the combination of 10R/10R ( rs28363170) and A carrier ( rs393795), of the *SLC6A3* gene could reduce the risk of levodopa-induced dyskinesias (LIDs) during long-term therapy with levodopa (Purcaro et al., 2019). As we all know, LIDs are characterized by dramatically increased synaptic DA concentrations in brain (Fuente-Fernandez et al., 2004). Thus, *SLC6A3* rs393795 AA genotype might be involved in the pathophysiological mechanisms underlying gait disorders in PD by reducing DA concentrations in frontal cortico-basal ganglia motor regions, such as SMA and ITG, and eventually cause compensatory activation of visual regions including right ITG. However, decreased ReHo values in right ITG were found in HC-AA compared with HC-CA/CC. Without the effects of disease, we preferred that decreased ReHo values of right ITG in HC-AA might be mainly caused by genetic polymorphisms. However, the deeper mechanism remains to be studied.

There were other limitations of our research. First, our research sample size is relatively small, i.e., small number genetic subgroups in the PD and HC samples, which may limit our ability to further classify genotypes and explore minor brain functional alterations related to *SLC6A3* rs393795. Second, the minor allele frequencies (MAF) of rs393795 (C) was 0.44 in our study, in good agreement with previous Chinese studies (Shang & Gau, 2014; Chu et al., 2019). Because the genetic polymorphisms are frequently different among the races, additional studies further investigating the SNP in PD are needed to clarify its role in different ethnicities. Third, although we had taken some measures to reduce the pharmacological effects on neural activity, we had to admit that the long-term effect of dopaminergic agonists could not be ruled out. Fourth, our study was conducted when PD patients were at rest and without dopaminergic treatments, more realistic studies should be performed, such as during a task or dopaminergic treatments. Fifth, we only investigate *SLC6A3* rs393795 in the present study, additional further and deeper studies are needed, such as gene-gene interactions. Finally, although TMT, FOG-Q and TUG tests were used to measure gait in our study, they could not fully assess the subtle gait characteristics that might be affected in PD, therefore more detailed assessments should be applied in future.

## Conclusions

With imaging genetics, our findings suggested that *SLC6A3* rs393795 allelic variation might have a trend to aggravate the severity of gait disorders in PD patients by altering the SMA and IFG function, and ultimately result in compensatory activation of right ITG. It could provide us with a new perspective for exploring deeply genetic mechanisms of gait disturbances in PD.

##  Supplemental Information

10.7717/peerj.7957/supp-1 Supplemental Information 1 Imaging raw data of PD patients Each data indicates the ReHo map for every PD patient. Abbreviations: ReHo, Regional homogeneity; PD, Parkinson’s disease.

10.7717/peerj.7957/supp-2 Supplemental Information 2 Imaging data of HC subjects, clinical data of all individuals and other materials **Imaging data part 2** includes the ReHo maps for HC subjects. The **Clinical Data**worksheet includes all demographic and clinical characteristics of participants involved in our study.
Abbreviations: ReHo, Regional homogeneity; HC, healthy control.

## Additional Information and Declarations

### Data Availability

The following information was supplied regarding data availability: The raw data is available in the Supplemental Files.
