# Supplementary material for: Allelic variant in SLC6A3 rs393795 affects cerebral regional homogeneity and gait dysfunction in patients with Parkinson’s disease
Source: PeerJ. 2019 Nov 4;7:e7957. doi: 10.7717/peerj.7957 (PMC6836753; doi:10.7717/peerj.7957)
Supplement: Supplemental Information 2 — Imaging data part 2 includes the ReHo maps for HC subjects. The Clinical Dataworksheet includes all demographic and clinical characteristics of participants involved in our study. Abbreviations: ReHo, Regional homogeneity; HC, healthy control. [file peerj-07-7957-s002.zip › SupplementalFiles2/Description.docx]

We provide relevant materials and raw data in Supplemental Files 1 and 2. The **Clinical Data** worksheet includes all demographic and clinical characteristics of participants involved in our study. In addition, all imaging data (**Imaging Data part 1 and 2**) are provided in MAT format, which could be read in Matlab software. Our imaging data could be analyzed using Statistical Parametric Mapping (SPM8: http://www.fil.ion.ucl.ac.uk/spm/) and Resting-State fMRI Data Analysis Tookit (REST: <http://www.restfmri.net>). And demographic and clinical characteristics of participants could be studied using SPSS 20.0 statistical analysis software (SPSS Inc. Chicago, IL, USA).
